# Coffee consumption and the risk of malignant melanoma in the Norwegian Women and Cancer (NOWAC) Study

**DOI:** 10.1186/s12885-016-2586-5

**Published:** 2016-07-29

**Authors:** Marko Lukic, Mie Jareid, Elisabete Weiderpass, Tonje Braaten

**Affiliations:** 1 Department of Community Medicine, Faculty of Health Sciences, UiT - The Arctic University of Norway, NO-9037 Tromsø, Norway; 2 Department of Research, Cancer Registry of Norway, Institute of Population-Based Cancer Research, Oslo, Norway; 3 Department of Medical Epidemiology and Biostatistics, Karolinska Institutet, Stockholm, Sweden; 4 Genetic Epidemiology Group, Folkhälsan Research Center, Helsinki, Finland

**Keywords:** Coffee, Filtered, Instant, Boiled, Melanoma, Prospective cohort, Multiple imputation

## Abstract

**Background:**

Coffee contains biologically-active substances that suppress carcinogenesis *in vivo*, and coffee consumption has been associated with a lower risk of malignant melanoma. We studied the impact of total coffee consumption and of different brewing methods on the incidence of malignant melanoma in a prospective cohort of Norwegian women.

**Methods:**

We had baseline information on total coffee consumption and consumption of filtered, instant, and boiled coffee from self-administered questionnaires for 104,080 women in the Norwegian Women and Cancer (NOWAC) Study. We also had follow-up information collected 6–8 years after baseline. Multiple imputation was used to deal with missing data, and multivariable Cox regression models were used to calculate hazard ratios (HR) for malignant melanoma by consumption category of total, filtered, instant, and boiled coffee.

**Results:**

During 1.7 million person-years of follow-up, 762 cases of malignant melanoma were diagnosed. Compared to light consumers of filtered coffee (≤1 cup/day), we found a statistically significant inverse association with low-moderate consumption (>1–3 cups/day, HR = 0.80; 95 % confidence interval [CI] 0.66–0.98) and high-moderate consumption of filtered coffee (>3–5 cups/day, HR = 0.77; 95 % CI 0.61–0.97) and melanoma risk (*p*_trend_ = 0.02). We did not find a statistically significant association between total, instant, or boiled coffee consumption and the risk of malignant melanoma in any of the consumption categories.

**Conclusions:**

The data from the NOWAC Study indicate that a moderate intake of filtered coffee could reduce the risk of malignant melanoma.

**Electronic supplementary material:**

The online version of this article (doi:10.1186/s12885-016-2586-5) contains supplementary material, which is available to authorized users.

## Background

Malignant melanoma constitutes 1.6 % of cancer cases diagnosed among women worldwide, with an estimated 111,000 cases in 2012. The highest age-adjusted rates are found in Australia/New Zealand (30.5 per 100,000). Melanoma incidence rates in Northern and Western Europe are 15.4 and 12.8 per 100,000, respectively [1]. In recent years, Norway has seen a rise in the age-adjusted incidence rate of malignant melanoma: from 15.9 per 100,000 in 2004 to 21.8 per 100,000 in 2013. Malignant melanoma now constitutes 6.3 % of cases and is the fourth most common cancer among Norwegian women [2].

Coffee contains caffeine, as well as potentially anticarcinogenic components such as chlorogenic acid, kahweol, and cafestol [3, 4]. However, levels of these components depend on brewing method [5, 6]. Prospective studies on coffee consumption and malignant melanoma have shown conflicting results, ranging from no association [7] to a lower relative risk [8–10]. Recent meta-analyses of observational studies reported inverse associations, with pooled relative risks of melanoma among regular coffee drinkers compared to controls of 0.75 (95 % confidence interval [CI] 0.63–0.89) [11, 12], and 0.80 (95 % CI 0.69–0.93) [11, 12].

Coffee consumption in the Norwegian population is high [13]. Thus, in the present study we investigated the association between total coffee consumption and consumption of filtered, instant, and boiled coffee on the incidence of malignant melanoma in a prospective cohort of Norwegian women.

## Methods

### Study population

The Norwegian Women and Cancer (NOWAC) Study is a nationally representative prospective cohort initiated in 1991. Women aged 30–70 years were randomly selected from the Central Population Register and mailed an invitation to participate in the study along with a self-administered questionnaire [14], which collected information on lifestyle, diet, and health status. More than 172,000 women returned the questionnaires (overall response rate: 52.7 %) and written informed consent was obtained from all study participants. The NOWAC Study was approved by the Regional Committee for Medical Research Ethics and the Norwegian Data Inspectorate.

We used baseline data collected during three waves of recruitment into the NOWAC Study (1991–1992, 1996–1997, and 2003–2004). Our initial study cohort consisted of the 110,548 women who completed a version of the NOWAC questionnaire that included questions on both coffee consumption by brewing method (filtered, instant, boiled) and lifetime incidence of sunburn. We excluded women with prevalent cancer other than non-melanoma skin cancer, those who emigrated or died before the start of follow-up (*N* = 3934), and those who did not have information on all three brewing methods (*N* = 2534). Following these exclusions, our final study sample consisted of 104,080 women. Of these, 91,707 returned a follow-up questionnaire 6–8 years after baseline, from which updated information on coffee consumption was taken. The remaining women (*N* = 12,373) that were recruited in 2004, did not have follow-up information available, as the follow-up questionnaire was sent out to them after the present study ended.

### Assessment of coffee consumption and sunburns

Both baseline and follow-up questionnaires contained the same question on coffee consumption: How many cups of coffee did you usually drink of each type (filtered, instant, boiled) during the past year? However, the response options for this question were different in the two versions of the questionnaire that were used during the study period. In the first version of the questionnaire, the women could choose between: never/seldom, 1–6 cups/week, 1 cup/day, 2–3 cups/day, 4–5 cups/day, 6–7 cups/day, and ≥8 cups/day. In the second version they could choose between: never/seldom, 1–3 cups/month, 1 cup/week, 2–4 cups/week, 5–6 cups/week, 1 cup/day, 2–3 cups/day, 4–5 cups/day, and 6–10 cups/day. Moreover, 7467 women received a modified version of the questionnaire at follow-up, in which they were asked to report total coffee consumption only. Total coffee consumption was calculated as the combined consumption of all brewing methods, and women were categorized by total coffee consumption, and by consumption of filtered, instant, and boiled coffee as: light consumers (≤1 cup/day), low-moderate consumers (>1–3 cups/day), high moderate consumers (>3–5 cups/day), and heavy consumers (>5 cups/day). As the size of a cup was not specified in the questionnaire, we used 2.1 dl as a standard cup size [15].

Information on the number of sunburns women sustained during their lifetime was taken from the baseline questionnaire, which collected information on the number of sunburns for 10-, 15-, 25- or 30-year periods, or a combination of these, depending on the participants’ age when the questionnaire was completed and the version of the questionnaire. For those who reported sunburns in at least three time periods, we calculated the average number of sunburns per year. For participants that reported sunburns in two time periods or less, the average number of sunburns was set as missing.

### Cancer incidence, death, and emigration

The unique 11-digit personal identification number assigned to every legal resident in Norway was used to acquire information on cancer incidence, death, and emigration in the cohort through linkage to the Norwegian Cancer Registry, the Cause of Death Registry, and the Norwegian Central Population Register, respectively. Cancer diagnoses were coded according to the 7^th^ Revision of the International Classification of Diseases, Injuries and Causes of Death. Code 190 was used to identify cases of malignant melanoma.

### Statistical methods

We used baseline information on coffee consumption until follow-up information became available, until date of diagnosis of any incident cancer other than non-melanoma skin cancer, death, or emigration, whichever occurred first. Once follow-up information became available, it was applied if the person remained in the study until diagnosis of any incident cancer other than non-melanoma skin cancer, death, emigration, or the end of the study period (31 December 2013), whichever occurred first [16]. We used Cox proportional hazards regression models to calculate hazard ratios (HRs) for developing malignant melanoma, with 95 % CIs, for each coffee consumption category. Light consumers (≤1 cup/day) were used as the reference group, as it was impossible to separate coffee abstainers and occasional coffee drinkers from the answers offered in the questionnaires. Attained age was used as the underlying time scale. All the models were stratified by questionnaire subcohort (i.e., recruitment in 1991–1992, 1996–1997, or 2003–2004) in order to control for potential differences in the long follow-up time.

We assessed the following potential confounders: average number of sunburns per year, original hair color, number of moles larger than 5 mm, and area of residence [17, 18]. We also assessed lifestyle factors that might confound the effect of coffee consumption on malignant melanoma: smoking status, education, body mass index (BMI), physical activity level, and alcohol consumption (g/day) [19–21]. The preliminary complete-case analysis included baseline information on total coffee consumption and potential confounders. As removal of any of these covariates led to a change in the regression coefficients of 10 % or more in each of the total coffee consumption categories, all of the potential confounders were retained as covariates in the final analyses of total coffee consumption and in brewing method-specific analyses. In addition, brewing method-specific analyses were adjusted for the two other brewing methods.

Due to the low number of cases in the highest consumption categories of boiled and instant coffee, we decided to conduct an additional analysis on the brewing methods. In the analysis, we dichotomized coffee consumption into “≤3 cups/month” (reference group), and “≥1 cup/week”.

We assessed linear trends by assigning a median value to each category of the ordinal coffee consumption variable, which was then modeled as a continuous variable in the analysis. We checked the proportional hazards assumption by visual inspection of the log-minus-log survival plot. Finally, we tested possible effect modification between coffee consumption and smoking status, BMI and average number of sunburns per year.

### Multiple imputation

In order to deal with missing information at baseline and follow-up, we performed multiple imputation under the assumption that data were missing at random. The missing values from baseline and follow-up were replaced by imputed values from 20 duplicate datasets that were created in order to reduce sampling variability from the imputation simulation [22].

All covariates used in the multivariable analyses were used as predictors in the imputation model. In addition, we used the Nelson-Aalen cumulative hazard estimator as a predictor along with the participants’ age at baseline in the imputation model [23]. Interaction terms between coffee consumption and smoking status or average number of sunburns were included as predictors in the imputation model if they were statistically significant in the complete-case analysis.

If total coffee consumption at follow-up was missing this value was not imputed, but calculated as the sum of the imputed values on consumption of filtered, instant, and boiled coffee. We imputed coffee consumption for the three brewing methods at follow-up for the 7467 women who received the version of the questionnaire that asked about total coffee consumption only.

The estimates from the twenty imputed datasets were combined using Rubin’s rules in order to obtain HRs and corresponding 95 % CIs [24]. All the analyses and the multiple imputations were done in STATA version 14.0 (Stata Corp, College Station, TX, USA).

## Results

During 1.7 million person-years of follow-up, there were 762 malignant melanoma cases. Mean follow-up time was 16.3 years. During follow-up there was an overall decrease in coffee consumption, mainly due to a drop in boiled and filtered coffee consumption (Table 1). The numbers of malignant melanoma cases across coffee consumption categories at the baseline are presented in Table 2.

**Table 1:** Distribution of participants according to total, filtered, instant, and boiled coffee consumption, the Norwegian Women and Cancer Study, 1991–2013 - complete case analyses

	Total coffee consumption			
	Light consumers	Low moderate consumers	High moderate consumers	Heavy consumers
	≤1 cup/day	>1–3 cups/day	>3–5 cups/day	>5 cups/day
Baseline, N (%)	16,853 (16.2)	29,923 (28.7)	34,451 (33.1)	22,853 (22.0)
Follow-up, N (%)	13,712 (19.4)	25,113 (35.5)	21,154 (29.9)	10,717 (15.2)
	Filtered coffee consumption			
	≤1 cup/day	>1–3 cups/day	>3–5 cups/day	>5 cups/day
Baseline, N (%)	41,942 (40.3)	26,708 (25.7)	23,014 (22.1)	12,416 (11.9)
Follow-up, N (%)	24,450 (38.3)	19,868 (31.2)	13,257 (20.8)	6179 (9.7)
	Instant coffee consumption			
	≤1 cup/day	>1–3 cups/day	>3–5 cups/day	>5 cups/day
Baseline, N (%)	95,584 (91.8)	5563 (5.3)	2070 (2.0)	863 (0.8)
Follow-up, N (%)	58,108 (91.1)	3941 (6.2)	1212 (1.9)	493 (0.8)
	Boiled coffee consumption			
	≤1 cup/day	>1–3 cups/day	>3–5 cups/day	>5 cups/day
Baseline, N (%)	83,094 (79.8)	8283 (8.0)	7474 (7.8)	5229 (5.0)
Follow-up, N (%)	56,441 (88.5)	3671 (5.8)	2258 (3.5)	1384 (2.2)

**Table 2:** Distribution of malignant melanoma cases according to total, filtered, instant, and boiled coffee consumption at baseline, the Norwegian Women and Cancer Study, 1991–2013

	Total coffee consumption	Filtered coffee consumption	Instant coffee consumption	Boiled coffee consumption
	n (%)	n (%)	n (%)	n (%)
Light consumers	134 (17.6)	305 (40.0)	627 (82.3)	691 (90.7)
≤1 cup/day				
Low-moderate consumers	234 (30.7)	218 (28.6)	57 (7.5)	45 (5.9)
>1–3 cups/day				
High-moderate consumers	239 (31.4)	160 (21.0)	51 (6.7)	16 (1.3)
>3–5 cups/day				
Heavy consumers	155 (20.3)	79 (10.4)	27 (3.5)	10 (2.1)
>5 cups/day				

The proportion of current smokers was lowest among light consumers and increased with higher coffee consumption, with 56.3 % of women drinking 5 or more cups of coffee/day being current smokers. There was a negative trend between total coffee consumption and duration of education, with an average of 13.1 years of schooling among light consumers and 11.1 years among heavy consumers. The average lifetime number of sunburns was similar across categories of total coffee consumption. Heavy consumers resided mainly in the northern and eastern part of the country (Table 3).

**Table 3:** Selected characteristics of the study sample by total coffee consumption at baseline, the Norwegian Women and Cancer Study, 1996–2013 - complete case analyses

Characteristics	Total coffee consumption			
	Light consumers	Low-moderate consumers	High-moderate consumers	Heavy consumers
	≤1 cup/day	>1–3 cups/day	>3–5 cups/day	>5 cups/day
Age at baseline (y), mean (SD)	47.8 (8.6)	49.0 (8.7)	47.4 (8.2)	46.3 (7.8)
Age at study exit (y), mean (SD)	63.4 (6.8)	64.6 (7.3)	64.1 (6.9)	63.5 (6.5)
Smoking status, %				
Never	50.8	42.4	31.6	17.1
Former	31.5	35.9	33.2	26.6
Current	17.7	21.7	35.2	56.3
Duration of education (y), mean (SD)	13.1 (3.6)	12.5 (3.5)	11.9 (3.3)	11.1 (3.1)
Body mass index, mean (SD)	24.0 (4.2)	24.0 (3.8)	23.9 (3.7)	24.0 (3.9)
Physical activity level, mean (SD)	5.5 (1.9)	5.7 (1.8)	5.7 (1.9)	5.5 (2.0)
Alcohol consumption (g/day), mean (SD)	3.0 (5.5)	3.4 (4.9)	3.3 (4.6)	3.2 (6.9)
Average number of sunburns per year, mean (SD)	0.9 (0.7)	0.8 (0.7)	0.8 (0.7)	0.8 (0.7)
Original hair color, %				
Dark	17.7	17.1	17.1	17.6
Brown	38.9	39.6	40.0	40.0
Blond	40.5	40.2	40.0	39.4
Ginger	2.9	3.1	2.8	3.0
Number of moles larger than 5 mm, %				
0	88.3	88.7	88.6	88.8
1	6.9	6.5	6.7	6.6
2–3	3.3	3.3	3.3	3.0
4–6	0.9	0.8	0.9	0.9
7–12	0.3	0.3	0.3	0.5
13–24	0.1	0.1	0.2	0.1
≥25	0.1	0.1	0.1	0.1
Area of residence, %				
Oslo area	14.7	10.2	7.2	5.3
Eastern Norway	39.9	36.2	34.8	30.7
Southern Norway	5.0	4.6	4.7	4.7
Western Norway	20.1	23.2	22.3	19.3
Middle Norway	6.7	7.7	8.2	9.0
Northern Norway	13.7	18.1	22.8	30.9

*y* years, *SD* standard deviation

The variables with the highest proportion of missing values at baseline were average numbers of sunburns (10.7 %), number of moles larger than 5 mm (10.0 %), and physical activity level (9.2 %). At follow-up, 22.9 % had missing values on total coffee consumption and 30.5 % had missing information on each brewing method. After multiple imputation, the characteristics of the study sample did not deviate substantially from the complete-case dataset (Table 4).

**Table 4:** Comparison of the complete-case dataset and the dataset with imputed values (multiple imputation), the Norwegian Women and Cancer Study, 1996–2013

Characteristics	Missing, N(%)	Complete-case	Multiple imputation
Total coffee consumption-baseline, mean (SD)	0 (0.0)	3.9 (2.5)	3.9 (2.5)
Total coffee consumption-follow-up, mean (SD)	21,011 (22.9)	3.4 (2.2)	3.4 (2.2)
Filtered coffee consumption-baseline, mean (SD)	0 (0.0)	2.6 (2.4)	2.6 (2.4)
Filtered coffee consumption-follow-up, mean (SD)	27,953 (30.5)	2.5 (2.2)	2.5 (2.2)
Instant coffee consumption-baseline, mean (SD)	0 (0.0)	0.3 (1.1)	0.3 (1.1)
Instant coffee consumption-follow-up, mean (SD)	27,953 (30.5)	0.4 (1.0)	0.4 (1.0)
Boiled coffee consumption-baseline, mean (SD)	0 (0.0)	0.9 (2.0)	0.9 (2.0)
Boiled coffee consumption-follow-up, mean (SD)	27,953 (30.5)	0.5 (1.4)	0.5 (1.4)
Smoking status, %			
Never	1595 (1.5)	34.6	34.7
Former		32.2	32.2
Current		33.1	33.1
Duration of education (y), mean (SD)	5184 (5.0)	12.1 (3.4)	12.0 (3.4)
Body mass index, mean (SD)	2379 (2.3)	24.0 (3.8)	24.0 (3.8)
Physical activity level, mean (SD)	9560 (9.2)	5.6 (1.9)	5.6 (1.9)
Alcohol consumption (g/day), mean (SD)	1686 (1.6)	3.2 (5.4)	3.2 (5.4)
Average number of sunburns per year, mean (SD)	11,103 (10.7)	0.8 (0.7)	0.8 (0.7)
Original hair color, %			
Dark	1587 (1.5)	17.3	17.3
Brown		39.7	39.6
Blond		40.0	40.1
Ginger		3.0	3.0
Number of moles larger than 5 mm, %			
0	10,444 (10.0)	88.6	88.7
1		6.6	6.6
2–3		3.2	3.2
4–6		0.9	0.9
7–12		0.4	0.4
13–24		0.1	0.1
≥25		0.1	0.1
Area of residence	0 (0.0)		
Oslo area		8.9	8.9
Eastern Norway		35.1	35.1
Southern Norway		4.7	4.7
Western Norway		21.5	21.5
Middle Norway		8.0	8.0
Northern Norway		21.8	21.8

*y* years, *SD* standard deviation

Compared to light consumers of filtered coffee, we found a statistically significant inverse association between low moderate (HR = 0.80; 95 % CI 0.66–0.98) and high moderate (HR = 0.77; 95 % CI 0.61–0.97) consumption of filtered coffee and the risk of malignant melanoma (*p*_trend_ = 0.02). There was a borderline, non-significant association between heavy consumption of filtered coffee and the risk of malignant melanoma (HR = 0.74; 95 % CI 0.53–1.02). We found no association between heavy consumption of boiled coffee (>5 vs ≤1 cup/day HR = 0.87; 95 % CI 0.49–1.55), instant coffee (>5 vs ≤1 cup/day HR = 1.45; 95 % CI 0.72–2.92), or heavy total coffee consumption (>5 vs ≤1 cup/day HR = 0.88; 95 % CI 0.67–1.14) and the risk of malignant melanoma. Similarly, no association was found when comparing the low-moderate and high-moderate consumption categories of total, instant, or boiled coffee with light consumption (Table 5). The risk estimates without adjustment for phenotypic and sun related factors are presented in the Additional file 1: Table S1.

**Table 5:** Hazard ratios (HRs) with 95 % confidence intervals (CI) of malignant melanoma (*n* = 762) according to total, filtered, instant, and boiled coffee consumption in the Norwegian Women and Cancer Study, 1991–2013 (*N* = 104,080)

	Total coffee consumption		Filtered coffee consumption		Instant coffee consumption		Boiled coffee consumption	
	Age-adjusted	Multivariable^a^	Age-adjusted	Multivariable^b^	Age-adjusted	Multivariable^b^	Age-adjusted	Multivariable^b^
	HR	HR	HR	HR	HR	HR	HR	HR
	95 % CI	95 % CI	95 % CI	95 % CI	95 % CI	95 % CI	95 % CI	95 % CI
Light consumers	1.00	1.00	1.00	1.00	1.00	1.00	1.00	1.00
≤1 cup/day								
Low-moderate consumers	0.93 (0.76–1.14)	0.95 (0.78–1.16)	0.85 (0.71–1.02)	0.80 (0.66–0.98)	1.34 (1.01–1.77)	1.17 (0.88–1.57)	1.01 (0.74–1.39)	1.13 (0.81–1.58)
>1–3 cups/day								
High-moderate consumers	0.77 (0.63–0.96)	0.85 (0.68–1.05)	0.77 (0.62–0.96)	0.77 (0.61–0.97)	0.92 (0.52–1.63)	0.85 (0.48–1.52)	0.74 (0.48–1.13)	0.89 (0.58–1.39)
>3–5 cups/day								
Heavy consumers	0.72 (0.56–0.92)	0.88 (0.67–1.14)	0.68 (0.50–0.92)	0.74 (0.53–1.02)	1.56 (0.78–3.10)	1.45 (0.72–2.92)	0.66 (0.38–1.14)	0.87 (0.49–1.55)
>5 cups/day								
*p*_trend_	0.002	0.20	0.002	0.02	0.10	0.39	0.07	0.72

*Cat.* categorical, *cont.* continuous

^a^Adjusted for smoking status, duration of education (cat.), body mass index (cat.), physical activity level (cont.), alcohol consumption (g/day) (cat.), area of residence, original hair color, number of moles larger than 5 mm (cat.), average number of sunburns per year (cont.)

^b^Adjusted for smoking status, duration of education (cat.), body mass index (cat.), physical activity level (cont.), alcohol consumption (g/day) (cat.), area of residence, original hair color, number of moles larger than 5 mm (cat.), average number of sunburns per year (cont.), and mutually adjusted for the consumption of coffee brewed with two other methods (cat.)

We did not find evidence of effect modification between coffee consumption and smoking status, BMI or average number of sunburns. After excluding melanoma cases diagnosed during the first year of follow-up and repeating the analyses for each of the brewing methods, the risk estimates were similar to those from the analyses that included the entire study sample (data not shown).

The analysis in which coffee consumption was dichotomized into “≤3 cups/month” and “≥1 cup/week” categories confirmed null findings from the main analysis regarding the association between instant and boiled coffee consumption and melanoma risk (Additional file 1: Table S2).

We conducted sensitivity analyses on different brewing methods for the 7467 women who received a version of the questionnaire that collected only information on total coffee consumption at follow-up. In these analyses, the baseline values were used throughout the study period. The effect estimates did not change compared to those obtained using imputed values (data not shown).

## Discussion

In this study, we added to the current knowledge on the effects of coffee consumption on the risk of malignant melanoma by including different coffee brewing methods (filtered, instant, and boiled). We found that a low moderate (>1–3 cups/day) or high moderate (>3–5 cups/day) intake of filtered coffee was associated with a decreased risk of malignant melanoma, accompanied with a statistically significant dose–response relationship. There was no association between instant, boiled, or total coffee consumption and melanoma risk.

Strengths of this study include the prospective design and a large sample size. Linkage to the Norwegian Cancer Registry allowed us to perform virtually complete follow-up of cancer cases. The external validation study indicates that the responders do not differ from the source population other than in somewhat higher educational level. The observed cumulated incidence rates for all cancer sites in the NOWAC study were almost identical to national figures [14, 25]. A 24-h dietary recall validation of the food frequency questionnaires used has shown a high validity of the information on coffee consumption (Spearman’s correlation coefficient *r* = 0.82) [15]. We used repeated measurements of coffee consumption in order to take into account changes in coffee consumption over time, which lowered the risk of measurement error. Finally, in order to maximize the number of participants, person-years, and melanoma cases included in the analyses, we used multiple imputation to deal with missing data.

There are also several limitations in the study.

While we had sufficient statistical power to detect differences between categories of total and filtered coffee consumption, the analyses of instant and boiled coffee were statistically underpowered. However, when we combined coffee consumption categories in order to increase the number of cases among the women who drank these coffee types, findings were similar to those from the main analysis. We decided to use women who drank ≤1 cup of coffee/day rather than “never/seldom” coffee consumers as the reference group, due to a low number of cases among “never/seldom” total coffee consumers. Moreover, seldom drinking or abstaining from coffee is relatively uncommon in Norway. Therefore, we believe that those women could differ from the women who reported drinking coffee more frequently, making them less appropriate as a reference group.

The information on certain types of coffee drinks, such as macchiato, espresso, cappuccino, or café latte, was not available from the questionnaires. Therefore, total coffee consumption may have been underestimated. However, consumption of such particular coffee drinks was uncommon among the women in the cohort at the time of data collection. Similarly, information on caffeination status was not available from the questionnaires. However, the consumption of decaffeinated coffee is uncommon in Norway. Hence, the measure of total coffee consumption used in the analyses was likely not substantially different from the true overall coffee intake. Tea consumption was not taken into account in the analyses, as this information was not available from all the NOWAC questionnaires. Tea contains some of the same anticarcinogenic components as coffee, and we cannot exclude a confounding effect of tea consumption. Finally, although we adjusted for many known risk factors, residual confounding cannot be completely ruled out.

Although a study confirmed the validity of the information on coffee consumption in the NOWAC questionnaires, misclassification is still possible. By using follow-up information on coffee consumption, we tried to reduce within-person variation and minimize the risk of misclassification bias. Nevertheless, misclassification due to a measurement error at both baseline and follow-up cannot be excluded, as information on coffee consumption was self-reported. Lifetime number of sunburns is a variable that cannot be validated. As such, retrospective reporting of sunburns over a period of decades may be only a rough estimate of the truth.

Information on ethnicity is not available in the NOWAC Study. If coffee drinking prevalence differed by ethnicity in the cohort, this could have been an unmeasured confounder. However, previous studies on sun exposure and melanoma in the NOWAC cohort concluded that hair color is a good indicator of sensitivity to sun exposure [18, 26]. Moreover, the percentage of migrants participating in the NOWAC study is likely very low, given the very low prevalence of foreign born women in the population at the time when the cohort was enrolled, and the fact that the questionnaires were all only available in Norwegian language.

We imputed missing information at baseline and follow-up, assuming a missing-at-random mechanism. All of the variables used in the main analyses were included in the imputation model. However, it is possible that at least some of the information was not missing at random, which would result in the obtained estimates not being completely free of bias. Finally, there were 7467 women who received a version of the questionnaire that only collected information on total coffee consumption at follow-up. For some of these women, the imputed values of the three brewing methods did not add up to the total coffee consumption they reported. However, when we conducted the analyses using the baseline values on different brewing methods for these women throughout the study period instead of imputing, the effect estimates were not different from those obtained using imputed values.

To our knowledge, this is the first study examining the effect of filtered, instant, boiled and total coffee consumption on the risk of malignant melanoma that used repeated information on coffee intake and combined this method with multiple imputation of missing data.

Evidence of no association between total, filtered, and boiled coffee intake and melanoma risk were found in the Swedish Västerbotten Intervention Project (VIP) involving both men and women [27]. The study had considerably fewer cases (*n* = 108), hence insufficient statistical power in order to detect weak associations. In two smaller studies in Norwegian women, both Stensvold and Jacobsen [28], and Veierød et al. [10] found a strong inverse association between heavy coffee consumption (≥7 cups/day) and melanoma risk (relative risk = 0.3, 48 cases; incidence rate ratio = 0.4, 61 cases, respectively). These studies, however, used “≤2 cups/day” as the reference group, in addition to a less extensive sun exposure adjustment. Furthermore, no information of brewing methods were presented in either of the studies. If filtered coffee was the most common brewing method among the participants, the results from these studies would reflect our findings.

A protective effect of caffeinated coffee consumption on melanoma risk was also found in the considerably larger National Institutes of Health-AARP Diet and Health (NIH-AARP) prospective cohort study (≥4 cups/day vs none HR = 0.80; 95 % CI 0.69–0.93). Despite not presenting results by gender, the authors reported that there was no significant heterogeneity in the results between men and women [8]. In the US, filtered coffee is predominant compared to other brewing methods [29], so it can be argued that the results from the NIH-AARP study are in line with our results. Similar findings were reported in the Nurses’ Health Study and Nurses’ Health Study II, which utilized updated information on coffee consumption throughout the follow-up period, and reported a 24 % risk reduction in women who drank >2 cups of coffee/day compared to non-coffee drinkers (95 % CI 0.64–0.89) [9]. Their risk estimates were similar to the estimates of filtered coffee consumption in the present study, despite the differences in the way of handling missing data at follow-up between these studies. In contrast, coffee consumption was not associated with risk of melanoma in the Women’s Health Initiative – Observational Study cohort of 66,484 postmenopausal women [7]. Finally, in a recent meta-analysis of two case–control and five cohort studies, Liu et al. reported a pooled relative risk of malignant melanoma of 0.81 (95 % CI 0.68–0.97) comparing the highest and the lowest quantity of caffeinated coffee intake [30].

Most experimental research on coffee constituents and skin cancer has been done on non-melanoma skin cancer, and there is clearly a need for mechanistic studies on the possible causal link between coffee and malignant melanoma. Caffeine, chlorogenic acid, cafestol, kahweol, and melanoidins are the most researched coffee constituents in relation to health [4], and have shown a range of anticarcinogenic effects in lab studies [4, 31]. We found an inverse association with filtered coffee, which rules out cafestol and kahweol as antimelanogenic compounds, since the content of diterpenes in filtered coffee is very low [5].

In UVB-induced non-melanoma skin cancer in mice, topical administration of caffeine induces an apoptotic response [32], and oral administration of caffeine inhibits the increase of cytokines responsible for the UVB-induced inflammatory response, which is thought to contribute to carcinogenesis, an effect similar to, and more effective when combined with, voluntary exercise [33]. The effect could be due to the positive effects of decreased body fat, or the fact that reduced subcutaneous fat restricts the energy available to skin tumors [34]. In a four-week intervention study in humans, filtered coffee consumption decreased body fat [35] and chlorogenic acid-enriched coffee decreased the expression of the inflammation marker interleukin 6 (*IL6*) [36]. We did not observe a difference in BMI or physical activity across categories of total coffee consumption.

The hallmarks of melanogenesis have been defined [37], and coffee contains compounds that target all of these hallmarks [31]. However, there seems to be little overlap in the pathways. Expansion of this multitarget functional perspective on coffee would be interesting. Little is known about the bioavailability of coffee components and coffee metabolites in human blood, and the physiological function of antioxidants and chemopreventive compounds in the diet [4], and this could be included in further studies using questionnaire data and blood samples.

## Conclusion

In the NOWAC Study, moderate consumption of filtered coffee is associated with a decreased risk of malignant melanoma. We found no evidence of an association between instant, boiled, or total coffee consumption and the risk of malignant melanoma.

## Abbreviations

BMI, body mass index; CI, confidence interval; HR, hazard ratio; NOWAC, Norwegian women and cancer; UVB, ultraviolet B

## Additional file

Additional file 1:
**Table S1.** Hazard ratios (HRs) with 95 % confidence intervals (CI) of malignant melanoma (*n* = 762) according to total, filtered, instant, and boiled coffee consumption in the Norwegian Women and Cancer Study, 1991–2013 (omitted adjustment for phenotypic and sun related factors, *N* = 104,080). **Table S2.** Hazard ratios (HRs) with 95 % confidence intervals (CI) of malignant melanoma (*n* = 762) according to, filtered, instant, and boiled coffee consumption with ≤3 cups/month as the reference cut-off in the Norwegian Women and Cancer Study, 1991–2013, *N* = 104,080. (DOCX 16 kb)
