# Supplementary material for: Coffee consumption and the risk of malignant melanoma in the Norwegian Women and Cancer (NOWAC) Study
Source: BMC Cancer. 2016 Jul 29;16:562. doi: 10.1186/s12885-016-2586-5 (PMC4966737; doi:10.1186/s12885-016-2586-5)
Supplement: Additional file 1: — Table S1. Hazard ratios (HRs) with 95 % confidence intervals (CI) of malignant melanoma (n = 762) according to total, filtered, instant, and boiled coffee consumption in the Norwegian Women and Cancer Study, 1991–2013 (omitted adjustment for phenotypic and sun related factors, N = 104,080). Table S2. Hazard ratios (HRs) with 95 % confidence intervals (CI) of malignant melanoma (n = 762) according to, filtered, instant, and boiled coffee consumption with ≤3 cups/month as the reference cut-off in the Norwegian Women and Cancer Study, 1991–2013, N = 104,080. (DOCX 16 kb) [file 12885_2016_2586_MOESM1_ESM.docx]

**Additional file 1: Table S1: Hazard ratios (HRs) with 95% confidence intervals (CI) of malignant melanoma (n=762) according to total, filtered, instant, and boiled coffee consumption in the Norwegian Women and Cancer Study, 1991-2013 (omitted adjustment for phenotypic and sun related factors,** **N=104,080)**

|  | **Total coffee consumption** | **Filtered coffee consumption** | **Instant coffee consumption** | **Boiled coffee consumption** |
| --- | --- | --- | --- | --- |
|  | **Multivariable^1^** | **Multivariable^2^** | **Multivariable^2^** | **Multivariable^2^** |
|  | HR  95% CI | HR  95% CI | HR  95% CI | HR  95% CI |
| **Light consumers**  **≤1 cup/day** | 1.00 | 1.00 | 1.00 | 1.00 |
| **Low-moderate consumers**  **>1-3 cups/day** | 0.94  (0.77-1.14) | 0.81  (0.67-0.99) | 1.17  (0.88-1.57) | 0.97  (0.70-1.35) |
| **High-moderate consumers**  **>3-5 cups/day** | 0.82  (0.66-1.02) | 0.78  (0.61-0.98) | 0.83  (0.46-1.47) | 0.74  (0.48-1.15) |
| **Heavy consumers**  **>5 cups/day** | 0.83  (0.64-1.07) | 0.74  (0.54-1.02) | 1.46  (0.73-2.94) | 0.70  (0.40-1.24) |
| *p*_trend_ | 0.08 | 0.02 | 0.43 | 0.12 |

^1^Adjusted for smoking status, duration of education (cat.), body mass index (cat.), physical activity level (cont.), alcohol consumption (g/day) (cat.) ^2^Adjusted for smoking status, duration of education (cat.), body mass index (cat.), physical activity level (cont.), alcohol consumption (g/day) (cat.), and mutually adjusted for the consumption of coffee brewed with two other methods (cat.).

Cat.: categorical; cont.: continuous.

**Additional file 1: Table S2: Hazard ratios (HRs) with 95% confidence intervals (CI) of malignant melanoma (n=762) according to, filtered, instant, and boiled coffee consumption with ≤3 cups/month as the reference cut-off in the Norwegian Women and Cancer Study, 1991-2013,** **N=104,080**

|  | **Number of cases** | **Filtered coffee consumption^1^** | **Number of cases** | **Instant coffee consumption^1^** | **Number of cases** | **Boiled coffee consumption^1^** |
| --- | --- | --- | --- | --- | --- | --- |
|  |  | HR  95% CI |  | HR  95% CI |  | HR  95% CI |
| **≤3 cups/month** | 189 | 1.00 | 590 | 1.00 | 612 | 1.00 |
| **≥1 cup/week** | 573 | 0.87  (0.72-1.06) | 172 | 1.14  (0.94-1.39) | 150 | 1.16  (0.94-1.42) |

^1^Adjusted for smoking status, duration of education (cat.), body mass index (cat.), physical activity level (cont.), alcohol consumption (g/day) (cat.), area of residence, original hair color, number of moles larger than 5 mm (cont.), average number of sunburns per year (cont.), and mutually adjusted for the consumption of coffee brewed with two other methods (cat.). Cat.: categorical; cont.: continuous.
